# Correlative 3D Imaging and Microfluidic Modelling of Human Pulmonary Lymphatics using Immunohistochemistry and High-resolution μCT

**DOI:** 10.1038/s41598-019-42794-7

**Published:** 2019-04-23

**Authors:** Stephanie K. Robinson, Jonathan J. Ramsden, Jane Warner, Peter M. Lackie, Tiina Roose

**Affiliations:** 1 Bioengineering Sciences Research Group, School of Engineering, Faculty of Engineering and Physical Science, University of Southampton, SO17 1BJ Southampton, England; 2 Clinical and Experimental Sciences, Faculty of Medicine, Southampton General Hospital, University of Southampton, SO16 6YD Southampton, England

**Keywords:** Biomedical engineering, Computational models, Anatomy

## Abstract

Lung lymphatics maintain fluid homoeostasis by providing a drainage system that returns fluid, cells and metabolites to the circulatory system. The 3D structure of the human pulmonary lymphatic network is essential to lung function, but it is poorly characterised. Image-based 3D mathematical modelling of pulmonary lymphatic microfluidics has been limited by the lack of accurate and representative image geometries. This is due to the microstructural similarity of the lymphatics to the blood vessel network, the lack of lymphatic-specific biomarkers, the technical limitations associated with image resolution in 3D, and sectioning artefacts present in 2D techniques. We present a method that combines lymphatic specific (D240 antibody) immunohistochemistry (IHC), optimised high-resolution X-ray microfocus computed tomography (μCT) and finite-element mathematical modelling to assess the function of human peripheral lung tissue. The initial results identify lymphatic heterogeneity within and between lung tissue. Lymphatic vessel volume fraction and fractal dimension significantly decreases away from the lung pleural surface (*p* < *0*.*001*, *n* = *25 and* p < *0*.*01*, *n* = 20, respectively). Microfluidic modelling successfully shows that in lung tissue the fluid derived from the blood vessels drains through the interstitium into the lymphatic vessel network and this drainage is different in the subpleural space compared to the intralobular space. When comparing lung tissue from health and disease, human pulmonary lymphatics were significantly different across five morphometric measures used in this study (p ≤ 0.0001). This proof of principle study establishes a new engineering technology and workflow for further studies of pulmonary lymphatics and demonstrates for the first time the combination of correlative μCT and IHC to enable 3D mathematical modelling of human lung microfluidics at micrometre resolution.

## Introduction

Pulmonary lymphatics act as a drainage system returning fluid, cells and essential metabolites to the circulatory system, thus maintaining fluid homoeostasis in the lung. Correct tissue fluid balance is critical for normal function as fluid accumulation in pulmonary oedema can lead to respiratory failure^1^. This balance is often disturbed in lung diseases such as Chronic Obstructive Pulmonary Disease (COPD) and lung cancer^2^. The bulk peripheral lymph drainage in the lung is believed to occur in the direction of the pleural surface within lymphatics before returning via the interlobular septae to the bronchopulmonary nodes and subsequently draining into major lymphatic trunks^3^. The microfluidic drainage of the pulmonary interstitium is poorly understood, especially within the interalveolar septae^1^.

The pulmonary lymphatics also provide conduits for rapid transport of lymphocytes and cell signalling molecules through regionally organised networks. The immune system cannot function normally if the lymphatics are absent^4^. Advances in oncology and autoimmunity have also highlighted the clinical interest in fully understanding the cellular transport within the lymphatic system^5^, especially in pathological conditions^6^. This, therefore, gives the rationale for an effective method to analyse and quantify microscale fluid dynamics within the human lung. A number of primary lymphatic drainage models exist^7–9^, however none to our knowledge model pulmonary-specific image based geometries through finite element (FE) mathematical modelling.

The recent identification of specific lymphatic biomarkers, coupled with the availability of antibodies for their visualisation, has enabled a greater understanding of the pulmonary lymphatic morphology and function^10,11^. Human lymphatic vessels have been shown by immunohistochemistry (IHC) to extend beyond the respiratory bronchioles, paralleling intralobular arteries deep inside the secondary lobule, with some being present and independent of blood vessels in the interalveolar septa^12^. The usefulness of LYVE-1, a widely used lymphatic endothelial marker, is limited in human tissue because of the variable performance of the antibody on routinely processed tissues^13^. Due to this, the majority of research undertaken on human tissue now favours anti-podoplanin antibodies (D240) as a superior lymphatic endothelial cell marker^10,12–14^.

The localisation of a tissue antigen by using IHC, once optimised, provides excellent image resolution although limited by 2D analysis which, when trying to characterise vessels, can be limiting. The process of sectioning for IHC is also destructive and sections can stretch and/or compress and generally deform the sample, which is problematic for morphometric studies^15^.

High-resolution X-ray micro-focus computed tomography (μCT) is a non-destructive, high-resolution imaging method that provides 3D structural detail of a sample with high fidelity. It has superior spatial resolution compared to more conventional diagnostic methods such as ultra-high-resolution computed tomography (U-HRCT) and magnetic resonance imaging (MRI)^16,17^. As soft tissue has low X-ray attenuation, lacks natural contrast and is difficult to fully immobilise, it has been historically challenging to image in 3D. Paraffin-embedded tissue provides the required mechanical tissue stability to obtain micro-focus radiographs whilst also enabling the use of hospital archived tissue which is linked to patient clinical data^15^.

Combining X-ray μCT and IHC provides a complete workflow allowing us to image, characterise and model the microfluidics of human pulmonary lymphatics and their associated structures at micrometre resolution in 3D. As far as we are aware this is the first work to combine correlative micro-scale imaging for use in 3D finite element microfluidic modelling within clinically linked human lung tissue.

Our group previously detailed a similar correlative imaging approach also using clinical data to characterise fibrotic plaques at micrometre resolution in patient lung samples with idiopathic pulmonary fibrosis^18^. This was achieved using Movat’s pentachrome staining to confirm the identity of features already visible in the X-ray CT dataset. Due to the vast numbers of well-established and highly specific antibodies now commercially available, the use of IHC furthers this approach. It allows highly specific identification of cellular features within the lung that would not otherwise be possible to discriminate in the X-ray μCT dataset.

Outside the use of human clinical tissue, there have recently been two publications by our group on the methods and the application of a similar workflow for microstructural 3D imaged-based mathematical modelling^19,20^. Synchrotron radiation-based computed tomography was used to image vasculature at micrometre-resolution to subsequently mathematically model oxygenation of murine skeletal muscle in 3D. The use of IHC in these particular studies was only used to confirm detail that was seen within the 3D data set rather than be used for additive detail undetected by the 3D imaging method as presented in the present study.

3D Image-based mathematical modelling of patient-specific flows, particularly in cardiovascular mechanics, has increased in recent years^21^. However, modelling fluid flow in 3D on clinically linked human soft tissue, to our knowledge, has not before been detailed to the microstructural level shown in this study. Our approach allows differences in 3D lymphatic morphometry between healthy and diseased lung samples to be identified and provides a mathematical simulation of how they function. This gives insight into how fluid imbalance arises in patients with pulmonary diseases.

## Materials and Methods

### Tissue Extraction and Preparation

Two peripheral lung tissue samples were obtained from two patients undergoing therapeutic resection surgery for lung cancer at Southampton University Hospital Trust (UK) with signed informed consent and full ethical approval from the National Research Ethics Service Committee, South Central—Southampton A, number 08/H0502/32. All methods were performed in accordance with the guidelines and regulations of the National Research Ethics Service Committee. Linked patient data from the sample referred to as ‘control’ had no indication of co-morbidities. The other sample referred to as ‘diseased’ was taken from a patient being treated with furosemide, suggesting fluid accumulation or hypertension. A sub-sample of around 1 cm^3^ for the control, and 0.6 mm^3^ for the diseased sample was taken at the periphery of the cancer resection to exclude tumour tissue, fixed in formalin for 48 hours and mounted in paraffin wax following routine histology protocols.

### µCT Scanning and 3D Image Reconstruction

**Figure 1:**
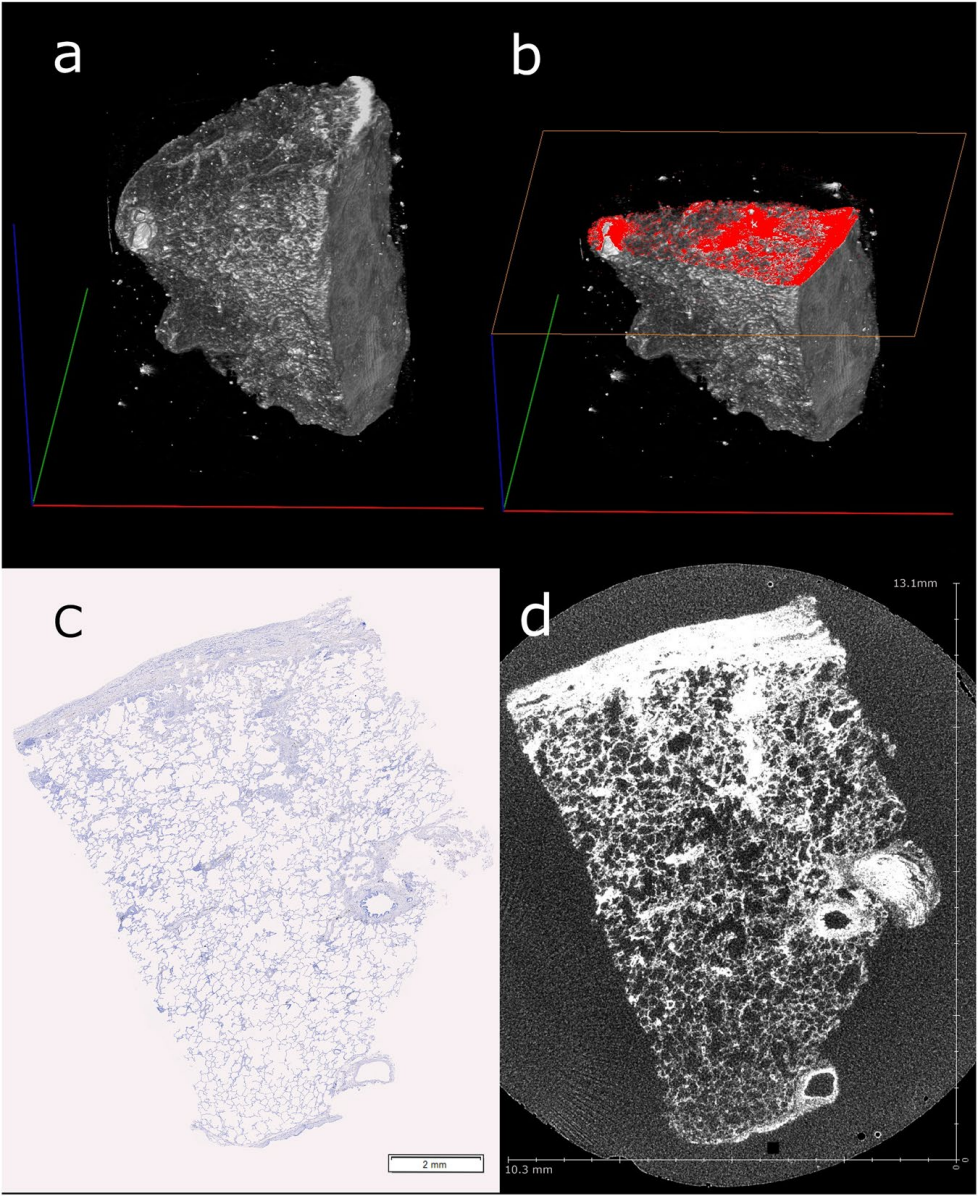
3D rendering and histological section alignment. (**a**) A 3D rendered image of the μCT scanned control human lung tissue block. Scale bars – Blue: 15.84 mm, Green and Red: 21.1 mm. (**b**) Image a plus an orthoslice section (red) located at the approximate position of the manual microtome sectioning. Blue: 12.2 mm, Green and Red: 21.1 mm. (**c**) The first of 20 serial stained tissue slices at 20x magnification. Distortion of tissue compared with image (**d**) can be seen width-wise. (**d**). An orthoslice view of the newly registered μCT data set to the histological slice.

A custom-built Nikon Metrology CT scanner (Nikon Metrology, Tring Herts, UK), with a 225 kV micro-focused X-ray source and a high sensitivity CsI scintillator, was used to scan the two samples. A Perkin- Elmer 1621AN flat panel detector recorded the X-ray penetration through the sample whilst being rotated. The resulting radiographs were digitally processed using a filtered-back-projection algorithm which generated a 32-bit float 3D volume file at a 13.2 µm voxel resolution for the control tissue (see Fig. 1a,b) and a 6 μm voxel resolution for the diseased sample. The increase in spatial resolution was due to the diseased sample being smaller. Technical details of the scanning protocol followed those of Scott *et al*.^15^.

### Tissue Sectioning and IHC

A 200 section series was cut on a paraffin microtome at a thickness of 5 µm for both lung samples. Every 10^th^ section was immunostained for lymphatic endothelial cells. This was achieved by using D240 (Abcam, Cambridge UK: ab77854) mouse monoclonal anti-human podoplanin primary antibody following a standard immunohistochemistry protocol. To confirm specific staining of the D240 antibody, anti-CD31 (Abcam: ab28364) and anti-pan-keratin (Sigma-Aldrich Company Ltd., Dorset, England: C-2562) were used to stain blood vessels and airways respectively on serial sections (see supplementary material page 1). Negative controls (omitting primary antibody) were included in each staining run.

An Olympus dotslide (Olympus, Southend-on-Sea, UK) system automatically obtained multiple images of the D240 stained slides at 20X magnification and digitised the resulting image as a VSI file. OlyVia software (Olympus) was used to view the slide images.

### Data Registration and Feature Segmentation

A 3D software platform; Amira 6.0, standard edition (FEI, Eindhoven, The Netherlands) was used to align the IHC section images to the corresponding μCT dataset using the slice module. This creates a digital μCT representation of the IHC section using reference features such as large bronchi or blood vessels. The μCT data set is then resampled in the orientation of the digital 2D slice using the Lanczos algorithm. This does not cause any elastic transformation of the μCT data set (see Fig. 1c,d).

**Figure 2:**
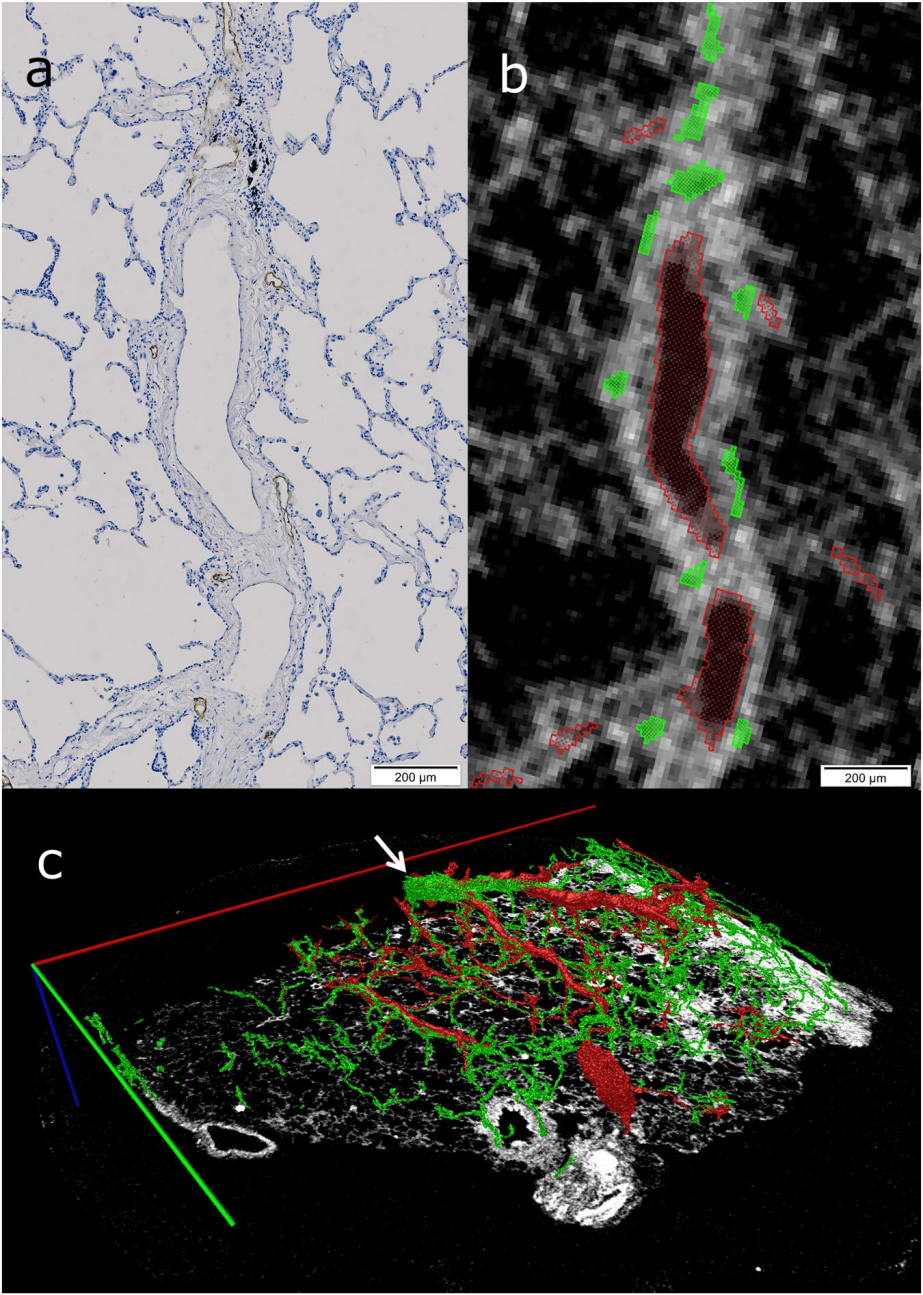
Feature segmentation. (**a**) A region of interest (ROI) of the histological slice showing the tissues stained in blue and the lymphatic vessels outlined in brown. A blood vessel is identifiable in the centre of the image by the dense connective tissue surrounding a smooth lumen. (**b**) The corresponding μCT ROI to image a with the segmented lymphatic vessels outlined in green and the blood vessel marked in red. (**c**) A rendered 3D image of segmented features within the μCT data set. Scale bars: red = 13.1 mm; green = 10.3 mm; blue = 0.5 mm) White arrow indicates the lymphatic vessel encircling a blood vessel. Lymphatics- Green, Blood vessels- Red, and Interstitial tissue –white.

Feature extraction was completed within Amira 6.0 across the whole tissue face in the X-Y plane to a depth of 500 μm in the Z direction (see Fig. 2) for both samples. The lymphatic vessel network was manually segmented by reference to the histology data (see Fig. 2a,b). The blood vessel network, excluding the capillary beds, was also manually segmented by use of µCT morphology (see Fig. 2b). Automatic thresholding was used to segment the whole of the lung tissue from alveolar spaces (Fig. 2c).

All segmented data were exported in a raw 3D format for subsequent multi-software use.

All lymphatic morphometric analysis and image processing was performed in the Fiji distribution of the ImageJ (version: 1.51 h) open-access software platform^22^. Morphometric analysis was undertaken on separate binary images made from the segmented lymphatic vessels, blood vessels and lung tissue from raw 3D data exported from Amira.

A script (macro language) was written in Fiji to determine sampling regions of interest (ROIs) within the X-Y plane of the lung tissue. This script randomly defined the coordinates of 25 and 20 ROIs of size 830 × 830 μm for the control and diseased sample respectively. This was within the full extent of each lung tissue on a selected Z-plane by use of a time-seeded random number generator. Any ROIs that overlapped were excluded and the script automatically reiterated up to a limit of 500 iterations. The sampling size was chosen to be representative of the 3D lymphatic network pattern (see Lymphatic Morphometry).

The ROIs were numbered and their distance from the 3D pleural surface was defined using a 3D 32-bit pixel distance map, taking the median pixel average within each of the ROIs. Any ROI that had an average pixel distance of less than 830 μm was labelled as being subpleural, and all others as being intralobular.

### Lymphatic Morphometry

The ROIs were extended through the Z-stack by 500 μm into 3D volumes of interest (VOIs) with dimensions of 830 × 830 × 500 μm each. These were used to define matching VOIs in the lymphatic vessel and tissue segmented data for both samples. A VOI refinement study indicated that VOIs above this size in both samples showed no variance in the lymphatic network pattern.

The volume and surface area of the lymphatic vessels, and the volume of the tissue was measured by the geometrical shape measure tool of the “3D ImageJ Suite” plugin version 2.7^23^. The lymphatic volume fraction (lymphatic volume/tissue volume) and lymphatic surface area/tissue volume ration were then calculated. The fractal dimension of lymphatic vessels present in each VOI was then measured using the BoneJ Fiji plugin version 1.4.2^24^. Finally the lymphatic branch number, junction number and tortuosity (branch length/Euclidean branch length) was measured by the “analyse skeleton” tool in Fiji after applying the “skeletonisation” algorithm to the lymphatic vessels within each VOI. These analyses were carried out for both the control and diseased samples. The Mann-Whitney U statistical test was performed to compare the 6 lymphatic morphometric measures between the control and diseased samples.

Finally the lymphatic volume fraction and fractal dimension of the control tissue were assessed in relation to the VOI distance from the pleural surfaceand subsequently, statistical regression analysis was performed for each parameter. We record here that 6 VOIs from the control tissue were excluded from the fractal dimension analysis due to only containing small partial volumes of vessels, causing the algorithm to fail.

### Image Processing for Mathematical Modelling

Two VOI were randomly chosen for finite element microfluidic modelling; one subpleural VOI; one intralobular VOI from the control tissue sample. Both VOIs underwent the following image processing. The VOI was extended in the Z-direction by re-segmentation to make a cubic VOI with a dimension of 830 × 830 × 830 μm. Separate images of the same VOI were created for each of the individually segmented features: Lymphatics; blood vessels and whole lung tissue. The 3 sub-volumes’ pixel dimensions were increased by a factor of 3 in each orientation so that 1 voxel was then represented by 9 voxels. This did not alter the geometry of the VOI. A median filter, averaging a grid of 9 cubic voxels, was then applied to the 3 images to smooth the images, necessary for FE mesh creation. Data which were not continuous with the lymphatics and blood vessels within the VOI were removed. To avoid voxel overlap within the VOI when the images were later combined, the lymphatic vessel image was subtracted from the blood vessel image using the image calculator tool in Fiji. The resulting blood vessel image and the lymphatic vessel image were then subtracted from the lung tissue image, leaving the lung tissue image representing only the alveolar walls, extracellular protein matrixes and the vasculature capillary beds; from this point on, this will be referred to as the interstitium. The resulting images were then exported as raw 3D images for FE meshing.

### Finite Element Mesh Generation

Scan IP (Simpleware, Exeter, UK) was used to make a mask of the edited lymphatic, blood vessel and interstitial tissue images for both VOIs separately. Within Scan IP, an FE model was then configured by creating a 3D mesh volume of all three masks assembled. The compound mesh coarseness was set to a value of 0 within a range from −50 to +50 as defined by a mesh refinement study. The interlobular VOI was meshed again with a scaling factor of +2 applied. This was created to assess the modelling effects of tissue shrinkage from formalin fixed paraffin embedded tissue preparation. In worse case scenarios this has been predicted to be as great as 50% hence the imaged VOI was increased by a factor of 2^25^. The export type was set as a COMSOL model volume geometry.

### Mathematical Model Implementation and Simulation

The mathematical model design, construction, and simulation were performed in COMSOL Multiphysics version 6.2, (COMSOL Ltd, Cambridge, UK) on a Windows operating system with an Intel(R) Xeon(R) CPU E5-2687W 0 @ 3.10 GHz, 16 core specification.

**Figure 3:**
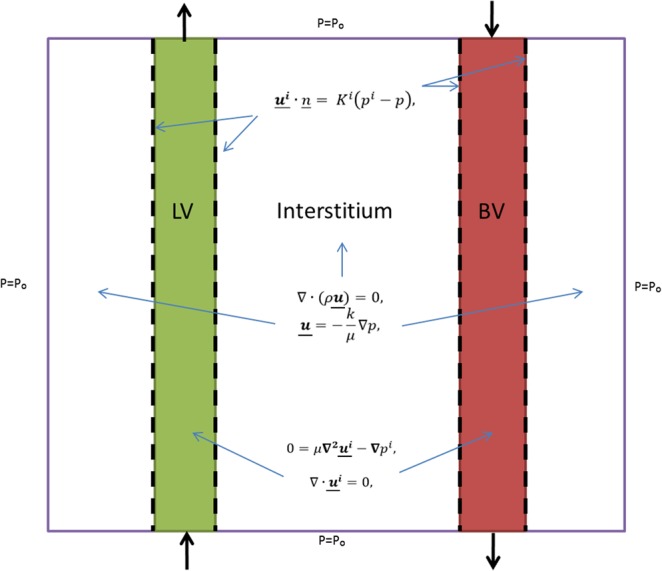
Schematic Representation of the Model in 2D for Pulmonary Fluid Flow. The blood vessel is shown in red (BV), the lymphatic vessel in green (LV) and the interstitial tissue in white. The vessels are described by Stokes’ flow and the interstitial tissue by Darcy’s Law. The dotted lines represent a flux boundary condition has been applied. Constrains of the model are shown by purple boundaries where an interstitial pressure condition was applied. All other symbols and initial parameter inputs are given in the text.

A 2D schematic representation of the mathematical model for pulmonary fluid flow is shown in Fig. 3. All input parameters used were taken from the relevant literature (see Table 1)

**Table 1:** Showing the input parameters for the lung tissue microfluidic mathematical model. All values have been quoted from the latest published literature.

Domain	Parameter	Value	Units	Description	Literature Source
Fluid	*μ*	1.2e^−3^	Pas	Dynamic Viscosity	^34^
	*ρ*	1030	Kg.m^−3^	Mass Density	^35^
Interstitium	*k*	4.4e^−18^	m2	Permeability	^35^
	*φ*	0.13	n/a	Porosity	^36^
	*p*	−1064	Pa	Pressure (interstitial)	^37^
lymphatics	*u* ^*L*^	3e^−4^	ms^−1^	Input velocity (normal to surface boundary)	^38^
	*p* ^*L*^	−1200	Pa	Pressure	^32^
	*K* ^*L*^	1.9e^−12^	*ms* ^*−1*^ *Pa* ^*−1*^	Hydraulic conductivity	^32^
Blood vessels	*u* ^*B*^	3e^−3^	ms^−1^	Input velocity (normal to surface boundary)	^39^
	*p* ^*B*^	+2000	Pa	Pressure	^39^
	*K* ^*B*^	2.71e^−12^	*ms* ^*−1*^ *Pa* ^*−1*^	Hydraulic conductivity	^32^

The fluid in the interstitial domain was modelled as incompressible using Darcy’s law that links the fluid flux $\underline{{\boldsymbol{u}}}$ to interstitial pressure gradient *p*:

1 $$\underline{{\boldsymbol{u}}}=-\,\frac{k}{\mu }\nabla p,$$

2 $$\nabla \cdot (\rho \underline{{\boldsymbol{u}}})=0,$$

where $\underline{{\boldsymbol{u}}}$ is the interstitial fluid velocity (m.s^−1^), *k* is the interstitial permeability, *p* is the lung interstitial fluid pressure (Pa), and *µ* and *ρ* are the dynamic viscosity (Pa.s) and mass density (kg.m^−3^) of the fluid respectively. A pressure boundary condition was applied to all the external interstitial surfaces of the model. Gravitational effects on the flow were neglected.

The fluid within the lymphatic and the blood vessel domains was described as a Newtonian fluid by the Navier-Stokes Equations with the inertial terms neglected. As this is consistent with a low Reynolds number regime (in the order of 10^−4^) we used Stokes flow^7^. The fluid flow is described as incompressible and laminar, thus, can be described in the form:

3 $$0=\mu {\nabla }^{{\bf{2}}}{\underline{{\boldsymbol{u}}}}^{{\boldsymbol{i}}}-\nabla {p}^{i},$$

4 $$\nabla \cdot {\underline{{\boldsymbol{u}}}}^{{\boldsymbol{i}}}=0,$$

where the superscript indexes *i* = *L* and *i* = *B* refer to lymphatic vessel and blood vessel values, respectively, and $\underline{{\boldsymbol{u}}}$ is the velocity vector. A no-slip boundary condition was applied at the vessel walls.

The fluid inputs at the lymphatic vessel and blood vessel boundaries were set as fluid velocity (m.s^−1^) normal to the boundary surface. As lymph drainage and blood flow are thought to occur in the direction of the pleural surface, the vessel boundaries positioned on the 3 VOIs faces furthest away from the pleural surface were selected as input boundaries. Within the intralobular VOI there was one boundary into the lymphatic vessel domain, and four boundaries into the blood vessel domain. Within the subpleural VOI there were six boundaries into the lymphatic vessel domain and three into the blood vessel domain. The vessel pressures defined the output condition which was applied to the remaining external boundaries on both vessel domains. Any fluid backflow was suppressed at these outlet boundaries.

A flux boundary condition was applied to each of the internal vessel-interstitium boundaries as described by:

5 $$\underline{{{\boldsymbol{u}}}^{{\boldsymbol{i}}}}\cdot \underline{n}={K}^{i}({p}^{i}-p),$$

where *i* = *L* and *i* = *B* refer to lymphatic vessel and blood vessel quantities respectively, ${\underline{{\boldsymbol{u}}}}^{i}\cdot \underline{n}$ is the volumetric flux (m^3^ m^−2^ s^−1^) normal to the vessel surface and *K (ms*^*−1*^ *Pa*^*−1*^) is the hydraulic conductivity constant specific to each vessel wall.

A direct linear solver was used to resolve these equations at equilibrium for the 3D meshed lung geometries with three dependent variables: Pressure, *p* (Pa); lymphatic fluid velocity field, ${\underline{{\boldsymbol{u}}}}^{{\boldsymbol{L}}}$ (ms^−1^); blood vessel fluid velocity field, ${\underline{{\boldsymbol{u}}}}^{{\boldsymbol{B}}}$ (ms^−1^) as this model is time independent.

To test the impact of the simulation results dependency on input parameters, the intralobular VOI geometry was used to perform a parameter sensitivity test. The lymphatic and blood vessel hydraulic conductivity constants, *K*^*L*^ *and K*^*B*^, and the lymphatic vessel domain input velocity, ${\underline{{\boldsymbol{u}}}}^{{\boldsymbol{L}}}$, was increased and decreased by 100x the values taken from the literature (see Table 1). These parameters were chosen to be investigated as they have the least experimental evidence supporting the proposed literature values. The lymphatic vessel flow velocity was taken from lymphatic vessels in the human skin as there is no experimental technique of measuring flow velocity within the internal lung lymphatic capillaries. There are estimates of lymphatic capillary flow velocities inferred from trunk flow rates and lymphatic vessel number that are two orders of magnitude smaller than the flow velocity of lymphatic vessels in the skin^26^. However, as the true numbers of lymphatic vessels are unknown these estimates are ambiguous. The hydraulic conductivity constants are usually calculated from measurements taken *in vitro*, or upon models that do not necessarily reflect *in vivo* conditions. Therefore, these values are also highly debated within the literature for blood vessels and especially for lymphatics.

### Ethics

Full ethical approval for the use of human samples of lung with informed consent was granted by the National Research Ethics Service Committee, South Central—Southampton A, number 08/H0502/32.

## Results

### Microstructural Imaging and Feature Segmentation

The D240 antibody successfully immunostained the lymphatics present in all sectioned lung tissues with no cross reaction to blood vessels or airways (see supplementary material). The antibody dilution of 1:80 was found to give optimal immunostaining.

The μCT scan data clearly differentiated the interstitial and pleural lung tissue from paraffin filled air spaces, permitting automated segmentation through thresholding of the lung tissue (see Fig. 1). The majority of pulmonary blood vessels had an open lumen and a greater surrounding density, thus, with the ability to trace anatomical structures through the data set in three different planes, blood vessel segmentation was achieved down to a diameter of 30 μm for the control tissue and 22 μm for the diseased tissue without the need for IHC.

Despite the lymphatic vessels having a similar density to the interstitial tissue, the lymphatic vessels were easily identified with the use of IHC. This allowed them to be segmented within the 3D μCT data set (see Fig. 2).

### 3D Lymphatic Morphometric Analysis

**Figure 4:**
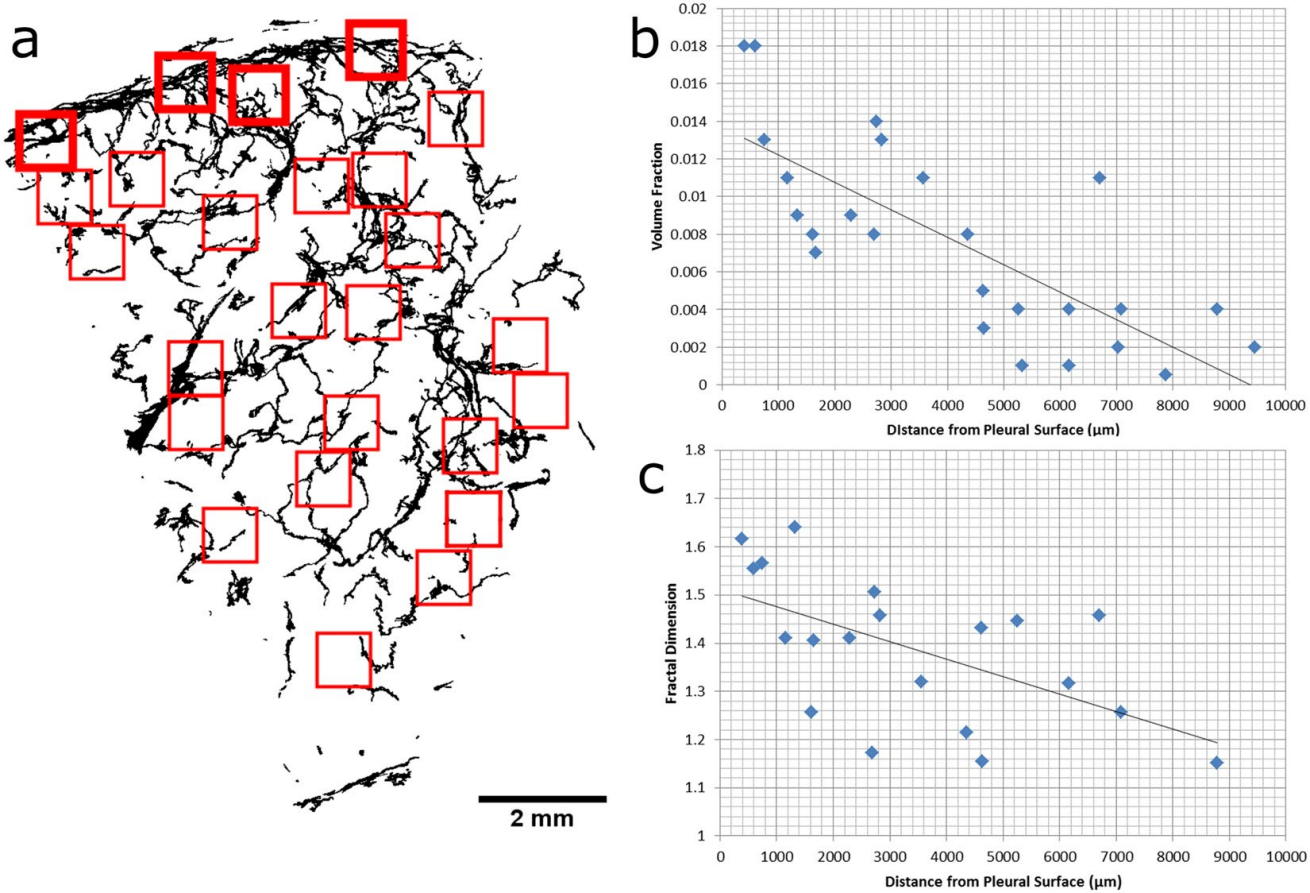
Spatial Morphometry of Pulmonary lymphatics. (**a**) The output of the ROIs given from macro-script are shown in red. The background data represents the binary image produced from the Z-stack maximum projection of the lymphatic segmentation of the healthy lung tissue. (**b**) The volume fraction of 25 lymphatic VOI plotted against their distance from the pleural surface of the lung tissue. (**c**) The fractal dimension of 20 VOI and their distance from the pleural surface of the lung tissue.

Out of the 25 VOIs generated for the control tissue, 4 were denoted as being at the pleural surface (subpleural interstitium) (bold on Fig. 4a), and the other 21 were labelled as interlobular interstitial VOIs. Out of the 20 VOIs generated for the diseased tissue, 9 were denoted as being at the pleural surface (subpleural interstitium), and the other 11 were labelled as interlobular interstitial VOIs.

**Figure 5:**
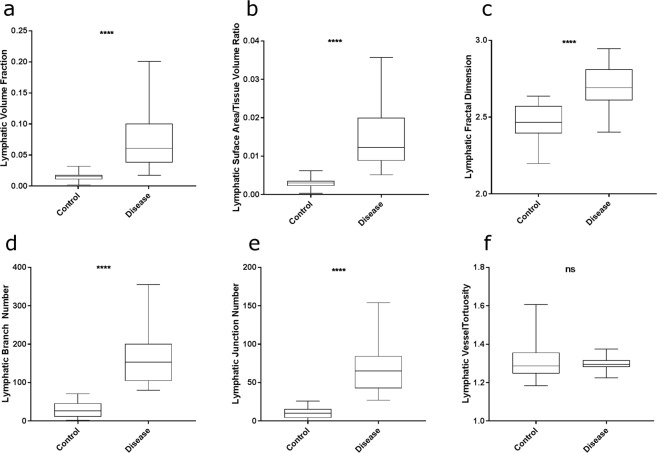
Quantitive morphology of pulmonary lymphatics between control and diseased tissue. A box and whisker plot of the lymphatic volume fraction (**a**) surface area/tissue volume ratio (**b**) fractal dimension (**c**) branch number (**d**) junction number (**e**) and tortuosity (**f**) between the control and diseased tissue. **** = p ≤ 0.0001. ns = not significant. Whisker plots show full range (min-max).

Lymphatic vessels were present throughout both lung tissue samples. There was a significant difference (p ≤ 0.0001) in lymphatic morphometry in 5 of the 6 measures between the control and diseased lung tissue samples (see Fig. 5). There was no significant difference in the tortuosity of the lymphatic vessels between the two samples.

In the control tissue, a significant inverse correlation existed between the volume fraction of lymphatic vessels within each VOI and increasing distance from the lung pleural surface (*n* = 25, *r* = *0*.*77*, *p* < *0*.*001)*. Also, a significant inverse correlation was found between lymphatic vessel fractal dimension and increasing distance from the pleural lung surface (*n* = 20, *r* = 0.58, *p* < 0.01); see Fig. 4b,c.

The intralobular lymphatics were predominantly associated with small blood vessels less than 40 μm in diameter, but were also observed occasionally on their own within the interalveolar septae. The subpleural lymphatics were less frequently associated with blood vessels. There was one perivascular lymphatic vessel that fully encircled the adjacent blood vessel (see Fig. 2c). Three of these encircling perivascular lymphatics were observed in the diseased tissue.

### Image Processing and FE Mesh Generation

The scaling and the subsequent median filtering step sufficiently smoothed the tissue images for the successful meshing of both VOIs. The image subtraction of the lymphatic and blood vessel binary images from the lung tissue image resulted in only the structures such as extracellular matrix proteins, alveolar walls and capillary beds of the μCT data set remaining within the interstitium image.

The FE mesh of the intralobular VOI was successfully generated in 12 hours and 17 minutes with a total of 5567408 tetrahedral elements, 1468796 triangular elements and 17433 edge elements. The FE mesh of the intralobular VOI scaled by 2 was successfully generated in 15 hours and 34 minutes with a total of 46702112 tetrahedral elements, 9881695 triangular elements and 35272 edge elements. The subpleural VOI was successfully generated in 18 hours and 37 minutes with a total of 9573048 tetrahedral elements, 1189976 triangular elements and 27148 edge elements.

### Solution to Fluid Flow in Peripheral Human Lung

The 3D simulation of the intralobular VOI geometry took 45 h 48 minutes and 38 seconds to reach a solution using a computer with 130 GB of physical memory and 150 GB of virtual memory. The error estimates in the simulation for the dependent variables were: Pressure (Pa) *p*; lymphatic vessel velocity field, ${\underline{{\boldsymbol{u}}}}^{{\boldsymbol{L}}}$; and blood vessel velocity field (m.s^−1^), ${\underline{{\boldsymbol{u}}}}^{{\boldsymbol{B}}}$, were 2.6.e^−16^, 9.e^−4^, 3.6.e^−4^ respectively.

The 3D simulation of the intralobular VOI geometry scaled by a factor of 2 took 108 h 24 minutes and 45 seconds to reach a solution using a computer with 137 GB of physical memory and 166 GB of virtual memory. The error estimates in the simulation for the dependent variables were: Pressure (Pa) *p*; lymphatic vessel velocity field, ${\underline{{\boldsymbol{u}}}}^{{\boldsymbol{L}}}$; and blood vessel velocity field (m.s^−1^), ${\underline{{\boldsymbol{u}}}}^{{\boldsymbol{B}}}$, were 2.7.e^−16^, 8.7.e^−4^, 2.7.e^−4^ respectively.

The 3D simulation of the subpleural VOI geometry took 49 h 51 minutes and 17 seconds to reach a solution using 168 GB of physical memory and 182 GB of virtual memory. The error estimates in the simulation for the dependent variables were: Pressure (Pa) *p*; lymphatic vessel velocity field, ${\underline{{\boldsymbol{u}}}}^{{\boldsymbol{L}}}$; and blood vessel velocity field (m.s^−1^), ${\underline{{\boldsymbol{u}}}}^{{\boldsymbol{B}}}$, were 8.7.e^−17^, 6.6e^−5^, 5.4.e^−4^ respectively.

**Figure 6:**
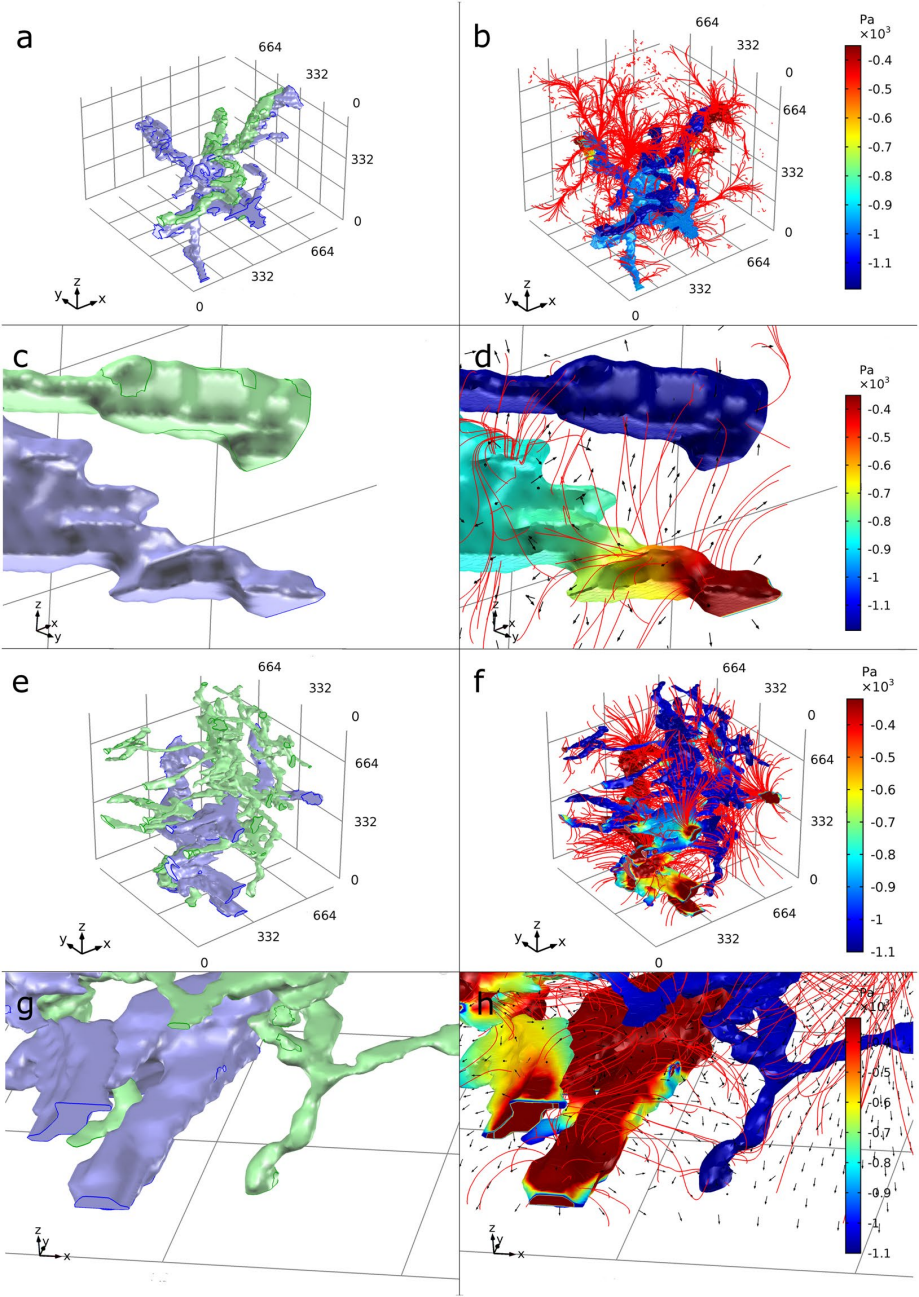
3D model simulation result for flow in an intralobular and subpleural lung geometry. A graphical representation for the control sample of the geometry and solution for the static pressure (Pa) within the lymphatic vessel and blood vessel of the intralobular VOI (**a** and **b** respectively), and subpleural VOI (**e** and **f** respectively). The interstitial pressure results have been removed from view so the streamlines could be visualised. (**c**,**d**,**g**,**h**) A close-up view of a part of images (**a**,**b**,**e**,**f)** respectively. Streamlines of the Darcy’s velocity field into the interstitium are shown in red. The direction of fluid flow is shown by black arrows. The VOIs in (**a**,**b**,**e**,**f)** have a dimension of 830 × 830 × 830 μm. In the geometry images the blood vessels = blue and the lymphatic vessel = green.

The Darcy’s velocity field streamlines and directional flow surface arrows indicate flow occurs from the higher pressured blood vessels through the interstitial tissue, in the case of the intralobular VOI circumventing the alveolar walls, into the lymphatic vessels (see Fig. 6). Not all fluid moved directly from the blood vessel to the lymphatic vessels, fluid also flowed to the distal regions of interstitial lung tissue. This was more apparent in the intralobular VOI compared to the subpleural VOI, where interstitial streamlines were shown to be longer, with fewer representing flow between blood vessels and lymphatic vessels (see Fig. 6b,f). When the identical model and parameters were applied to the scaled intralobular VOI, the streamlines of interstitial flux were identical, thus directional movement of fluid did not change. The quantity of fluid flux out of the blood vessel and into the lymphatic vessel did change however, due to the pressure gradient change as shown in Supplementary Figure 2. The streamlines of the fluid flow inside both vessels were complete and directional.

The parameter sensitivity test showed that with a 100 fold increase or decrease in either the lymphatic or blood vessel hydraulic conductivity constant, the absolute change in volumetric flux into the intralobular VOI lymphatic vessel domain (m^3^.s^−1^) at equilibrium was below the error limits of simulation. The absolute changes in volumetric flux with the change in lymphatic input velocity were above the error limits of simulation. With a 100 fold increase in the lymphatic input velocity, the percentage change in volumetric flux into the intralobular VOI lymphatic vessel domain (m^3^.s^−1^) at equilibrium was −82.7%. With a 100 fold decrease in the lymphatic input velocity, the percentage change in volumetric flux into the intralobular VOI lymphatic vessel domain (m^3^.s^−1^) at equilibrium was +0.8%.

## Discussion

In this paper we demonstrate that combining μCT with IHC allows for the unambiguous identification of small tissue components and the accurate representation of their 3D structure within the samples studied. This structure can then be taken forward for FE mathematical modelling.

Using IHC alone to attempt 3D tissue morphometrics has many limitations. Serial sections are rarely complete or of consistent thickness. In addition, sections can be significantly deformed due to microtome artefacts such as blade compression or stretching due to heating in a water bath. As μCT is not invasive and is non-destructive, we have shown it provides a robust reference structure that can be used to compensate for histological artefacts. Using paraffin-embedded samples provides sample stability and the opportunity to use archived collections of clinically-characterised human tissue previously biopsied and processed for routine histological analysis.

It may be possible to address the shrinkage artefacts of formalin fixation and paraffin embedding of soft tissue from animal studies. A μCT scan of tissue before and after sample preparation could be performed allowing a digital volume transform to be calculated. Non-uniform shrinkage artefacts could also be assessed this way rather than accounting for predictive uniform shrinkage artefacts as demonstrated in the current study. However, this was not possible in this study as we were dealing with human patient samples that were already archived and stored.

Obtaining the lymphatic vessel network in peripheral human lung tissue and using that structure to model the microfluidic flow of lymph was chosen for a proof of principle in this study. However, this workflow allows for a wide variety of antibodies, validated for formalin fixed, paraffin embedded IHC, to be used for 3D-specific analysis and subsequent mathematical modelling across many different tissue types in human and animal studies.

### Identifying a 3D Structure by Combining μCT and IHC

Some structural elements of the tissue could be directly identified in the μCT datasets. In both lung samples assessed, the μCT scan, which allowed tissue images to be viewed from all orientations, enabled the segmentation of blood vessels under 50 μm without the use of IHC on the grayscale morphology alone. The airways were also easily identified due to the collapsed star-like cross-sectional appearance. We do not anticipate that all lung tissue samples will have this clarity of structural detail, especially if the tissue is necrotic. In these cases, the use of IHC to confirm the identity of ambiguous structures may be necessary.

The lymphatics were difficult to visualise within the μCT dataset alone as the grey values represented them were too similar to the surrounding interstitial tissue with few vessel lumens apparent. The IHC sections confirmed some lymphatic vessels appeared to be in a partially collapsed or fully collapsed state supporting previous observations of lymphatic vessel structure^27^. Manual segmentation of stained features from IHC into a μCT scan relative to other structural features was essential for maintaining 3D lymphatic structure fidelity. At present automatic registration, segmentation and interpolation methods require both images to be at the same resolution. As the microlymphatic vessels have been shown here to be small and highly complex and any reduction in the IHC resolution required to visualise the stain would be lost thus rendering automated methods impossible. Due to this limitation, more advanced computational software for correlative imaging is presently in high demand, and we therefore expect that automated, or semi-automated soft tissue segmentation will be possible in the future.

The use of D240 antibody has previously been shown to be highly specific for lymphatic vessel identification in lung tissue^12,28^. Although the specificity of the immunoreactivity to the lymphatic vessels was not presented in this work, the D240 reactivity was comparable to that seen in previous work and was not that of blood vessels or airways (see supplementary material). Combining μCT and IHC demonstrated that the 3D vessel structure obtained from the D240 reactivity in the lung was well-defined with no unconnected structures giving evidence to support D240’s capability to specificity stain the lymphatic endothelium.

### Intra-sample Sampling

The inclusion of the pleural surface in all IHC sections was important as it has previously been reported that drainage of the lymph inside the lung tissue occurs towards the pleural surface^29^. Within this constraint, the sampling was randomised to avoid bias. The frequency of IHC sections stained for lymphatics using the D240 antibody was chosen to visualise all notable deviations in 3D morphology. The number and size of the VOIs used to assess the lymphatic morphology was selected to encompass the 3D patterns observed in the network. However, a systematic study to assess the effect of the sampling size and/or number for effective lymphatic morphometric analysis could be undertaken. The script that created the sampling VOI can be used to create any size and number of ROIs within a defined 2D area.

### Characteristics of Pulmonary Lymphatics

The distribution of the lymphatic vessels within both lung tissue samples was consistent with previous 2D pulmonary lymphatic IHC studies^12,28^. It was not possible to fully identify the type(s) of lymphatic vessel mapped with this methodology. However, based on size alone the majority of the vessels identified were microlymphatics; sometimes termed initial lymphatics or primary lymphatics (15 μm-75 μm diameter), with one or two collecting lymphatics; sometimes termed secondary lymphatics (35 μm–300 μm) being found only in the peribronchovascular tissue of the diseased samples^30^. Ultrastructural studies would give more evidence to determine the classification of the lymphatics present within the intralobular interstitium. The finding of a peribronchovasular lymphatic vessels fully encirculating blood vessels emphasises the importance of 3D structural morphometry in understanding pulmonary lymphatic function. This is an example of a feature that would not have been apparent from 2D studies.

As a proof of principle study, we describe the 3D pulmonary lymphatics with six morphometric measures; volume fraction; surface area/tissue volume ratio; fractal dimension; branch number; junction number and tortuosity. Characterising the pulmonary lymphatics with these measures all reflected the relative complexity of the lymphatic vessel structures which may or may not influence fluid flow, and can inform further study designs.

Assessing lymphatic morphometry on the acquired lymphatic structures will help to inform which VOIs to take forward for flow simulation to investigate if this particular geometrical variation influences fluid uptake by, or fluid flow within, the lymphatic vessel.

As the present study only used two human tissue samples as a proof of concept, further studies are required to make firm conclusions about inter- and intra-sample variation of lymphatic morphometry. However, the greater lymphatic volume at the subpleural interstitium compared to the intralobular interstitium supports the 2D IHC study that showed the proportion of area occupied by lymphatics was greatest in the subpleural interstitium compared to the intralobular interstitium^28^. The increase in the volume of lymphatics in the diseased sample compared to the control also supports a 2D study where patients with COPD had increased parenchymal lymphatics compared to the control^14^.

The greater fractal dimension of the lymphatics in the subpleural interstitium of the control tissue is a novel finding. Until further investigation confirms or contradicts this finding, it is impossible to comment further as to the reason behind this observation without being speculative. It gives another example of the importance of obtaining an unambiguous 3D structure of a tissue feature in morphometric studies.

The workflow presented will allow these initial findings to be fully investigated in future studies of human lymphatic morphology. Increasing the number of lung tissue samples and the number of separate individuals from which they have been obtained will be essential for validating these findings. Subsampling in the same tissue may also give insight into the presence or absence of structural variation within the same individual and how this may influence lymphatic vessel functionality. Ultimately this workflow could be applied to the comparison of the 3D lymphatic morphometry of healthy lung samples with diseased lung samples such as those from COPD and lung cancer patients or other pulmonary diseases such as Idiopathic Pulmonary Fibrosis. This may give insight into why fluid based symptoms arise in patients with these diseases.

To consider the variability of lymphatic structure and function across the whole organ, strategic sampling of the whole organ or lobules would be needed. This is certainly of interest and achievable in animal studies as whole organ or lobule harvesting has in this case fewer challenges than in human studies.

In future work, the use of animal studies would be of benefit to understand the limitation of using excised, collapsed lung compared to the “*in-situ*” lung. Lung could be inflated through negative pressure to a set physiological volume before fixation, and subsequently put through the workflow laid out in this study. This would help to understand temporal and dynamic external influences on lymphatic density and volume, currently a limitation of using fixed human tissue.

### FE Mathematical Modelling

Image smoothing is a necessary step for the successful generation of an FE mesh. This also mitigates small errors that may have occurred through image segmentation, yet it should be noted that it does not eliminate them. Scaling up the number of pixels in each image before applying the median pixel filtering step allowed filtering to be 3 times finer than it would be if applied to the original image thus minimising alterations to the original data. The volume of discontinuous vessel data removed was very small and unlikely to be significant. It should be noted that this processing was performed identically on the two VOIs of the same type of tissue.

The results obtained from the mathematical simulation of fluid flow are broadly in line with the known physiology that interstitial fluid bathes all cells within the lung to nourish and remove waste products from them. As the geometry was of human lung tissue that was not evidently diseased, and the input parameters used were as close to normal human physiological parameters as possible, it was encouraging to obtain the simulation results consistent with this physiology.

Simulating the flow in the Intralobular VOI that has been scaled by a factor of 2 highlights the importance of addressing the shrinkage artefacts of formalin fixation and paraffin embedding. Accounting for this shrinkage in the mesh geometry is a good comprise for archived tissue, yet direct measurement of shrinkage would be advised as described above. This was not possible in the present study as we were dealing with archived human tissue samples.

The results obtained from simulating two VOIs that had morphometrically different structures, highlights how the results obtained from the morphometric assessment of the presented workflow could be used to inform comparative modelling studies. Here we show qualitative differences in the direction and length of interstitial fluid flow between the two geometries, however, in the future we expect that sophisticated numerical analysis of these simulations may give support to functional differences in how these structures contribute to fluid drainage in the lung.

The parameter sensitivity tests demonstrated that changing the hydraulic conductivity of either vessel wall by 100 fold changed the volumetric flux by less than the simulation error. Increasing the lymphatic input velocity by 100 fold demonstrated an 82.7% decrease in volumetric flow into the lymphatic vessel due to a considerable reduction in the pressure gradient between the interstitium domain and the lymphatic vessel domain. Considering this higher lymphatic flow velocity is of the same order of magnitude as that measured in the canine thoracic duct, this change is academic, and the relevance of this to peripheral lung flow is small^31^.

These results would be expected in low pressured biological systems such as the lung as there is typically considerable scope for changes in physiological parameters in order to maintain homeostatic control. It should be noted however that this simulation was a representation of a small section of lung tissue, and when scaled up, even very small changes in volumetric flux could have an impact on the organ as a whole.

Overall, the modeling results support the concept that a change in the 3D image geometry is more important than the input parameters in its influence on fluid flow simulation within the lung.

As a proof of principle study, we recognise the model’s limitations. The novelty of this paper lies in the advancement of obtaining the real geometry and length scales for modelling rather than in the sophistication of the mathematical model. The lung capillary bed was modelled as a porous media domain within the interstitial tissue by Darcy’s law. This assumption has been seen in non-imaged based models of the lung and although not ideal; it is argued that the model is still representative as long as values for interstitial tissue porosity are altered to take this into account^32^. As the vasculature represented within the model is not of capillary size, and based on the known anatomy of the secondary lobule, the segmented vascular structures are likely to be an arteriole in the case of the intralobular VOI and a venule in the case of the subpleural VOI. In addition to this, no cellular components were taken into account when modelling flow in the blood and lymphatic vessel; hence Stokes’ flow is suitable in this model for the obtained geometries. As no variation in wall thickness is apparent in our 3D images of the vasculature, we can only assume that the permeability of these vessels throughout the example VOI is constant. As the capillary beds are known to be extremely dense in the lung, we do not anticipate this simplification will impact significantly on the model’s accuracy; however, further analysis is needed to confirm this.

Currently, the interstitial porosity is modelled as constant throughout the geometry. In reality, the interstitial tissue is made up of large numbers of extracellular matrix proteins and glycoproteins. All of these are of varying density, arranged differently and retard fluid at differing rates. It is not clear what impact these tissue constituents have on fluid flux, but it may be important. Building on the work presented here, it will be possible to use the methods similar to previous work carried out in our group where an image based model for fluid flow within a mouse lymph node was created that considered the hydraulic conductivity to be dependent on the pixel brightness in the image of the interstitium^33^.

Finally, the dynamic influence of cyclic strain on fluid flow has not been included in this model due to the model being a proof of principle example. However, the importance of cyclic strain cannot be underestimated, especially in the lung.

### Future Development of the Model

To our knowledge, this is the first work to model any 3D microfluidic flow in image-based human lung geometries, and as a proof of concept example, it is hoped that the presented workflow will be used to address the aforementioned limitations. This could include the addition of imaging the capillary network by use of IHC. Although manual segmentation of these structures in the lung would be time intensive, it would give a better representation of the direct motion of fluid from the arterioles into the interstitium rather than assuming Darcy’s law across the capillary bed. Also, using Starling’s law to model precisely the movement of fluid from the blood vessels into the interstitial tissue would better reflect the current understanding of tissue microfluidics.

Once the model is more biologically detailed (when this detail becomes quantified), one could advance the sensitivity tests performed in this study to identify in finer detail the importance and optimal values of the various input parameters used in the model. If the geometry being used is that from a healthy individual, this could be extremely useful in understanding the extent of the physiological function in human lung tissue. In addition, if this workflow was applied to tissue samples from patients with a full clinical history, the lymphatic functionality within and between patients with varying clinical conditions could be determined. The influence of differences in lymphatic and associated lung geometrical structures on the rate or volume of fluid clearance in the lung could provide important insights into the pathogenesis of human lung disease.

As mentioned above, using animal models, lung tissue could be obtained at different stages of the breathing cycle through negative pressure inflation and put through the workflow presented in this paper. The dynamic change of tissue volume could be used to incorporate dynamic cyclic strain into the model.

## Conclusions

In conclusion, we have presented a structured workflow that can be used to investigate the 3D structural morphology of the microstructures within a human lung sample and demonstrate how this structural information can be used for initial finite element microfluidic mathematical modelling. Although the results from using this workflow are preliminary, it demonstrates a clear application for this workflow for 3D morphometric studies and 3D mathematical modelling.

## Supplementary information

Supplementary Information

## Data Availability

All connected data to this manuscript will be made available through the following DOI (10.5258/SOTON/D0310).
