# Supplementary material for: Correlative 3D Imaging and Microfluidic Modelling of Human Pulmonary Lymphatics using Immunohistochemistry and High-resolution μCT
Source: Sci Rep. 2019 Apr 23;9:6415. doi: 10.1038/s41598-019-42794-7 (PMC6478691; doi:10.1038/s41598-019-42794-7)
Supplement: Supplementary file 1 — Supplementary Information [file 41598_2019_42794_MOESM1_ESM.docx]

Supplementary material for:

***Correlative 3D Imaging and Microfluidic Modelling of Human Pulmonary Lymphatics using Immunohistochemistry and High-resolution μCT***

Stephanie K. Robinson^1,2^; Jonathan J. Ramsden^2^; Jane Warner^2^; Peter M. Lackie^2^; Tiina Roose^1^.

1. Bioengineering Sciences Research Group, Faculty of Engineering and the Environment, University of Southampton

2. Clinical and Experimental Sciences, Faculty of Medicine, University of Southampton.

***Corresponding Author***

Miss Stephanie K Robinson, sr1g12@soton.ac.uk, 02381204807

***Immunohistochemistry protocol***

Sections were deparaffinised in a clearene solution and rehydrated through graded alcohols. A 0.016% hydrogen peroxide solution in methanol was then applied onto the sections for 10 minutes. The sections were then washed in tris-buffered saline (TBS) for 3 x 2 mins. Any pre-treatment necessary for primary antibody binding was then undertaken (see below for details). A pre-made avidin/biotin blocking kit (Vector Laboratories LTD, Peterborough, UK) was then applied with a 20 min application of both avidin and biotin with a 3 x 2 min wash in-between. The sections were then washed again for 3x2min before applying a blocking culture media for 20 mins made up with: Dulbecco’s modified eagles medium (Sigma); fetal calf serum (PAA, Cell Culture Company, Yeovil, UK); bovine serum albumin (Sigma). This media was then drained (not washed) and the primary antibodies at an optimised dilution (see below) were then applied to the sections overnight at 4°C.

The sections were allowed to reach room temperature then washed in TBS for 3x 5 mins. Biotinylated secondary antibodies were then applied to the sections for 30 mins. Goat anti-mouse (Vector: BA-9200) was used at a 1:800 dilution; goat anti-rabbit (Vector: BA-1000) was used at a 1:400 dilution. Sections were then washed in TBS for 3x 5 mins. A pre-made “ABC” complex of streptavidin (A), biotinylated horseradish peroxidase (B) (Vector) and TBS (1: 1: 75) was then applied to the sections for 30 mins before being washed in TBS for 3x 5 mins. A diaminobenzidiine (DAB) chromogen solution (BioGenex, Fremont, CA, USA), stable DAB buffer and a 15% sodium azide solution (3: 100: 5)was then applied to the sections for 5 mins and rinsed in TBS. Sections were run in tap water for 5 mins. Sections were then counterstained with Mayer’s haematoxylin for 20 seconds and ‘blued’ under running tap water for 5 mins and subsequently dehydrated through graded alcohols before being cleared with clearene. Sections were then mounted with Pertex.

***D240 antibody cross-reactivity assay***

Three serial lung sections were individually stained by following the above protocol. The primary antibodies used were D240 (abcam:ab77854), anti-CD31 (abcam:ab38264) and anti-pan-keratin or anti-pan-cytokeratin (PCK) (Sigma-Aldrich:c-2562) to visualise the lymphatics, blood vessels and airways respectively.

Anti-CD31 was used at a 1:100 dilution with a heat mediated pre-treatment in a 0.01M Citrate buffer solution (microwave at 50% power for 25 mins). PCK was used at a 1:4000 dilution with a pre-treatment of pronase (Dako, Agilent Pathology Solutions, Stockport, UK) in TBS (1:20) for 10 minutes. No tissue pre-treatment was necessary for staining with D240. The results of this assay showed that the D240 antibody did not stain airways or blood vessels. Based on the known morphology of lymphatics and the similarity of the stain to previous work (see main text), we conclude that the D240 antibody immunoreactivity represents specific binding to the lymphatic endothelium (see s-figure 1).

***
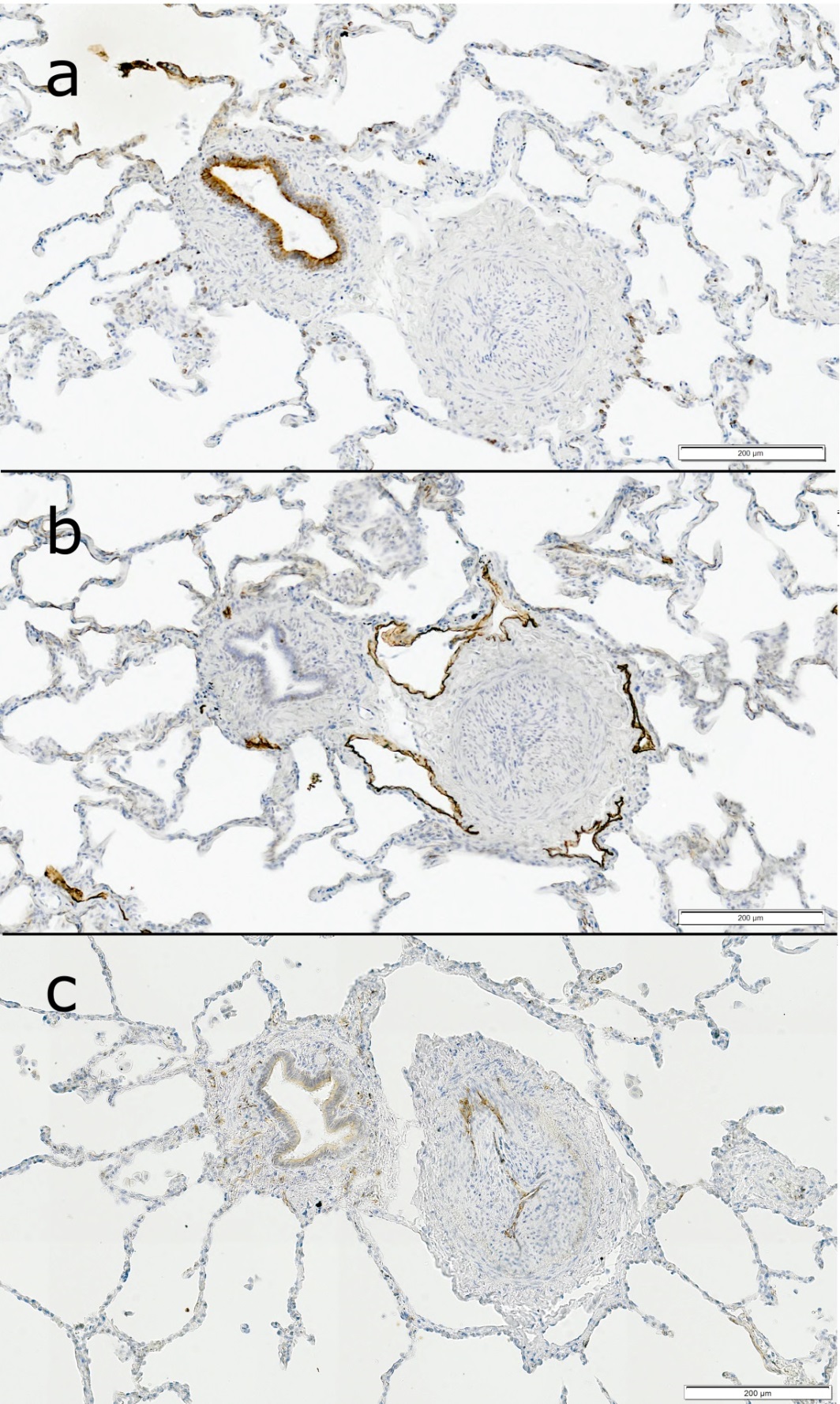
***

***S-Figure 1: D240 Cross-reactivity assay. a:*** *Immunostaining with PCK against the epithelial lining of the airways within a bronchovascular bundle of a healthy human lung tissue sample.* ***b:***  *Immunostaining using D240 against the lymphatics within a bronchovascular bundle.* ***c:*** *Immunostaining with anti- CD31 against the blood vessels (collapsed arteriole and capillaries) within a bronchovascular bundle.*

***
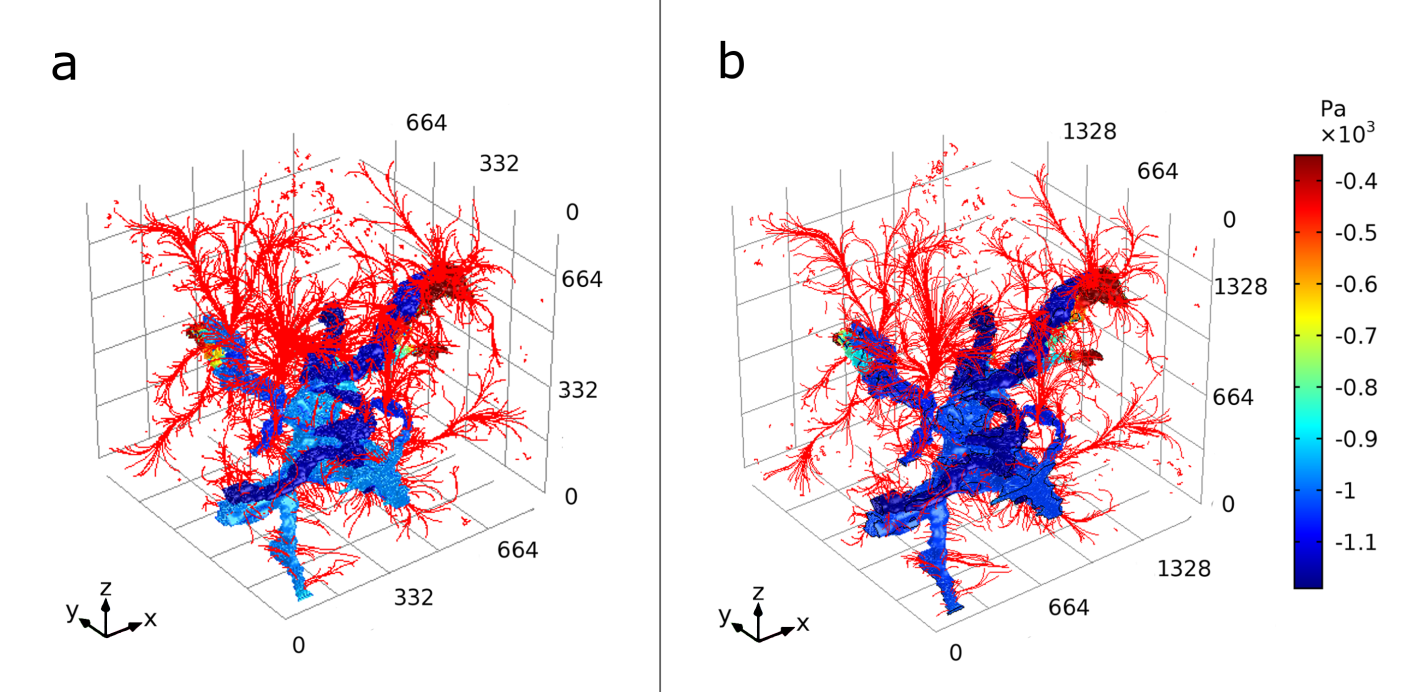
***

***S-Figure 2: Graphical Result of the Model Simulation to Assess the Influence of Shrinkage Artefacts.*** A graphical representation of the solution for the static pressure (Pa) within the lymphatic vessel and blood vessel of the control intralobular VOI at the imaged and measured length scale **(a)** and of the measured VOI increased by a scale factor of 2 **(b).** Streamlines of Darcy’s velocity field into the interstitium are shown in red. The geometries have dimensions of 830x830x830μm **(a)** and 1660x1660x1660μm **(b)**
